# Associations of Binge Drinking With the Risks of Ischemic Heart Disease and Stroke: A Study of Pooled Norwegian Health Surveys

**DOI:** 10.1093/aje/kwab063

**Published:** 2021-03-15

**Authors:** Eirik Degerud, Gudrun Høiseth, Jørg Mørland, Inger Ariansen, Sidsel Graff-Iversen, Eivind Ystrom, Luisa Zuccolo, Grethe S Tell, Øyvind Næss

**Keywords:** alcohol consumption, binge drinking, ischemic heart disease, prospective observational study, stroke

## Abstract

Norwegian health survey data (1987–2003) were analyzed to determine if binge drinking increases the risk of incident major events from ischemic heart disease (IHD) and stroke. Among current drinkers reporting average alcohol intakes of 2.00–59.99 g/day (*n* = 44,476), frequent binge drinking (≥5 units at least once per month) was not associated with a greater risk of IHD (adjusted hazard ratio (HR) = 0.91, 95% confidence interval (CI): 0.76, 1.09) or stroke (adjusted HR = 0.98, 95% CI: 0.81, 1.19), in comparison with participants who reported that they never or only infrequently (less than once per month) had episodes of binge drinking. Participants with an average alcohol intake of 2.00–59.99 g/day had a lower risk of IHD in comparison with participants with very low intakes (<2.00 g/day), both among frequent binge drinkers (adjusted HR = 0.67, 95% CI: 0.56, 0.80) and among never/infrequent binge drinkers (adjusted HR = 0.75, 95% CI: 0.67, 0.84). The findings suggest that frequent binge drinking, independent of average alcohol intake, does not increase the risk of incident IHD or stroke events. However, the findings should be interpreted in light of the limitations of the study design.

## Abbreviations

CI: confidence interval
CVD: cardiovascular disease
HDL-C: high-density lipoprotein cholesterol
HR: hazard ratio
IHD: ischemic heart disease
MI: myocardial infarction

There is a differential association between the average intake of alcohol over time and the risk of cardiovascular disease (CVD), according to data from observational studies (1). In conventional observational studies, the average intake of low to moderate amounts of alcohol has been associated with a lower risk of ischemic stroke (2), whereas high intake amounts have been associated more consistently with a higher risk of ischemic and hemorrhagic stroke (2, 3). In some genetically informed observational studies (4, 5), but not all (6), any alcohol intake was associated with a higher risk of both stroke subtypes. The genetically informed data are also consistent with a more pronounced increase in the risk of hemorrhagic stroke (4, 5) and with increased blood pressure as a likely mediator (5, 7).

In conventional observational studies, the risk of ischemic heart disease (IHD) has been consistently lower among drinkers in comparison with nondrinkers (3, 8, 9), with the exception of chronic heavy drinkers. However, genetically informed observational studies have not reproduced the protective association (4–6), indicating that the association could be a result of unmeasured confounding or exposure misclassification of “sick quitters” (10). Increased high-density lipoprotein cholesterol (HDL-C) and reduced fibrinogen levels have been proposed as mediators of a protective association, and although there are some recent data supporting a small causal effect from lowering fibrinogen levels (11), there is not support for a causal effect from increasing HDL-C levels (12, 13).

Binge drinking is the consumption of a large quantity of alcohol on a single occasion. This leads in the short term to intoxication and impairment of the central nervous system, which increase the risk of accidents and violence. Accordingly, the general population is advised to avoid binge drinking to keep health risks from drinking at a low level (14, 15). Frequent binge drinking (≥5 units at least once per month), also called heavy episodic drinking, is included in the World Health Organization’s definition of harmful alcohol use (16). The extent to which alcohol-related disease mechanisms are sensitive to binge drinking, independently of the average quantity of alcohol consumed over time, is not well known. There are also limited data on whether the long-term health risks associated with drinking a given quantity of alcohol over time, such as the risk of different CVDs, differ between people who tend to drink in binges and those who tend to spread alcohol intake over more days.

In this study, we report data on the association of binge drinking with the risk of CVD from a large sample of the Norwegian general population. We aimed to compare the risk of incident fatal and nonfatal IHD and stroke events between people who drink the same quantity of alcohol over time but differ in their tendency to binge drink. We hypothesized that the risk of both IHD and stroke at a given intake level is higher among people who tend to binge drink frequently in comparison with those who never or rarely binge drink. We also hypothesized that there is not a lower risk of IHD associated with alcohol intake among binge drinkers, which was the result of a meta-analysis of previous observational studies (17).

## METHODS

### Study population, data linkage, and selection

We selected current drinkers with no history of major IHD events or stroke (defined by self-report or a hospital record of a previous acute or old myocardial infarction (MI) or a previous stroke or stroke sequela, respectively) from Norwegian population-based health surveys and a survey from the Norwegian Twin Registry. The surveys (Web Table 1, available at https://doi.org/10.1093/aje/kwab063) were conducted between 1987 and 2003 and comprised 88,394 observations. The percentages of men and women were similar and age at attendance ranged from 19 to 89 (mean age, 52) years. Most individuals only responded to a single survey, but a small subset (approximately 3%) responded more than once. In this study, we only included data from 1 survey per individual. Priority between surveys was a trade-off between the quality of alcohol data and the length of follow-up, which is relevant to the number of observed outcomes. Missing data were handled using list-wise deletion. Data linkage and de-identification were performed by Statistics Norway.

### Alcohol exposure

Alcohol intake data were harmonized from the survey questionnaires (Web Table 1). Depending on the data available from each survey, we estimated the quantity of alcohol consumed on average (grams per day) from the total number of glasses (1 glass = 12.8 g of alcohol; wine, beer, and spirits were reported separately) consumed over a defined time, or by combining the average number of units consumed per occasion (0–20; higher values were truncated) with the drinking frequency. The latter estimation required the following conversions: infrequent drinking = 6 times per year, 1/month = 12 times per year, 2 – 3/month = 30 times per year, 1/week = 52 times per year, 2 – 3/week = 130 times per year, and 4 – 7/week = 286 times per year. The following intake categories were used in this study: <2.00, 2.00–11.99, 12.00–23.99, and 24.00–59.99 g/day. Data on frequency of binge drinking were obtained by asking participants, “How often do you drink the equivalent or more than 5 units per occasion.” In this study, we used a dichotomous variable differentiating between frequent binge drinkers who meet the definition of heavy episodic drinking (i.e., ≥5 units on a single occasion at least once per month) and those who never or infrequently binge drink (less than once per month).

### Covariates

Marital status (married vs. divorced/never married/widower) was obtained from the National Registry or from self-report. The National Educational Database provided data on the level of education attained until 2011 (range, 1–8, in which 1 equals primary school and 8 a master’s or doctoral degree). Data on current smoking (yes/no), physical activity (range, 1–4, in which 1 is sedentary and 4 very active), diabetes, and familial history of CVD were based on self-report. Body mass index (kg/m^2^), for all but 1 survey, was measured by study personnel. Serum HDL-C levels were measured from nonfasting venous blood samples and these data were available for a subset of participants.

### Outcome data and follow-up

A database of national hospitalization records (Cardiovascular Disease in Norway project, https://cvdnor.w.uib.no/) and The Norwegian Cause of Death Registry provided outcome data. The latter is based on physician death certificates and, occasionally, autopsy reports; the registry completeness is 98% of deaths among Norwegian residents domestic and abroad (18). The major IHD event end point was defined as the first occurrence of hospitalization with acute MI (*International Classification of Diseases, Ninth Revision* (ICD–9) code: 410; ICD–10 codes: I21, I22) as the main or secondary diagnosis, or death due to coronary heart disease as the underlying cause (1990–1995: ICD-9 codes: 410–414; 1996–2009: ICD-10 codes: I20- I25), whichever came first. The stroke event end point was defined as hospitalization with acute cerebral stroke (ICD–9 codes: 430–434, 436; ICD–10 codes: I60–I61, I63-I64, except I63.6) as the main or secondary diagnosis, or death due to cerebral stroke as the underlying cause, whichever came first. The use of main and secondary diagnoses in the national hospitalization records had sensitivity, specificity, and positive predictive values of 85.8%, 99.7%, and 95.1%, respectively, for acute MI (19), and 97%, 99.6%, and 79.7%, respectively, for stroke (20), compared with hospital records.

### Statistical analyses

Analysis of variance and the χ^2^ test were used to assess differences in descriptive statistics. Participants were followed from time of survey and until an incident event, emigration, death, or December 31, 2009. Cox models were used to estimate hazard ratios and 95% confidence intervals. To test the hypothesis that binge drinking is associated with a higher risk of IHD and stroke, we estimated hazard ratios comparing frequent and infrequent/never binge drinking among people drinking, on average, of 2.00–59.99 g/day, and among the following intake strata: 2.00–11.99, 12.00–23.99, and 24.00–59.99 g/day; as well as age and sex. People drinking less than 2 g/day were excluded to avoid biasing this analysis (a person had to drink, on average, ≥2.00 g/day to be defined as partaking in frequent binge drinking).

We also hypothesized that among frequent binge drinkers, there would not be a lower risk of IHD associated with their alcohol intake. To test this, we estimated hazard ratios for IHD and stroke according to average alcohol intake in the sample overall and stratified by binge drinking. Participants drinking less than 2.00 g/day on average were used as a common reference category and in combination with lifetime abstainers in a subgroup analysis (21).

The main analyses testing the 2 hypotheses were repeated in a larger sample that included one additional survey that did not have data on drinking quantity. In these analyses, drinking frequency, not drinking quantity, was used as the measure of overall alcohol intake. We also estimated hazard ratios for a 3-level categorical presentation of binge drinking. Cox models were adjusted for age and sex; a second multivariable model was adjusted in addition for education, marital status, smoking, physical activity, body mass index, and familial history of CVDs, which are confounders frequently adjusted for in observational studies (10). A third model was additionally adjusted for binge drinking or average drinking quantity, as appropriate. Analyses were conducted using the R programming language, version 3.4.2 (R Foundation for Statistical Computing, Vienna, Austria) in the integrated development environment of RStudio, version 1.1.383 (RStudio, Boston, Massachusetts).

## RESULTS

### Participants

The source population comprised 88,394 survey visits or observations nested among 85,677 individuals. Each individual contributed data from a single survey, resulting in the exclusion of 2,717 observations. We then excluded participants who did not drink alcohol (*n* = 11,136), were chronic heavy drinkers (*n* = 158), had missing values or who answered inconsistently on different questions about their alcohol intake (*n* = 13,945), who reported or had a hospital record of previous IHD or stroke events (*n* = 3,047), or who had missing values on covariates (*n* = 2,429). A sample comprising 54,962 participants with data on binge drinking and drinking frequency was available for additional analyses. The final study population with data on binge drinking and drinking quantity comprised 44,476 participants. The flow chart in Web Figure 1 provides more details.

### Descriptive statistics

The average drinking quantity was higher among men, smokers, and people with higher HDL-C levels, and was inversely associated with marital status (being married) and family history of coronary heart disease (Table 1). The relationships with education, physical activity, body mass index, and diabetes were nonlinear and, to some extent, characterized by more favorable values in terms of CVD risk prevention in the groups drinking 2.00–11.99 g/day and 12.00–23.99 g/day. Participants reporting frequent binge drinking were younger, more often men, less likely to be married, more likely to smoke, slightly more physically active, had a higher level of education, and drank more alcohol on average than individuals reporting never or infrequent binge drinking.

**Table 1:** Descriptive Statistics According to Categories of Average Drinking Quantity and Binge-Drinking Frequency Among Norwegian Adult Men and Women (*n* = 44,476) Who Participated in a Cardiovascular Health Examination Survey in Midlife and Reported to be Currently Drinking Alcohol, 1987–2003

**Binge-Drinking Frequency by Characteristic** ^**a**^		**Average Quantity of Alcohol Consumed, g/day**															***P* Value** ^**c**^
	**Total. No.**	**All (*n* = 44,476)**			**<2.00** ^**b**^ **(*n* = 16,332)**			**2.00–11.99 (*n* = 22,699)**			**12.00–23.99 (*n* = 4,367)**			**24.00–59.99 (*n* = 1,078)**			
		**No.**	**%**	**Mean (SD)**	**No.**	**%**	**Mean (SD)**	**No.**	**%**	**Mean (SD)**	**No.**	**%**	**Mean (SD)**	**No.**	**%**	**Mean (SD)**	
Age, years																	
All	44,476			49.7 (14.4)			51.3 (15.2)			48.5 (13.8)			49.7 (13.8)			50.3 (14.3)	<0.001
Never/infrequent	18,743			51.4 (13.9)						50.8 (13.8)			56.5 (12.9)			59.5 (13.0)	<0.001
Frequent	9,401			43.4 (12.2)						41.6 (11.3)			45.8 (12.7)			48.0 (13.6)	<0.001
*P* value		<0.001						<0.001			<0.001			<0.001			
Sex, male																	
All	44,476	23,287	52.4		6,820	41.8		12,487	55.0		3,087	70.7		893	82.8		<0.001
Never/infrequent	18,743	9,797	52.3					8,629	51.0		1,002	62.7		166	76.9		<0.001
Frequent	9,401	6,670	70.9					3,858	66.9		2085	75.3		727	84.3		<0.001
*P* value		<0.001						<0.001			<0.001			0.012			
Education, 1–8^d^																	
All	44,476			4.3 (1.8)			3.9 (1.7)			4.5 (1.7)			4.9 (1.8)			4.6 (1.8)	<0.001
Never/infrequent	18,743			4.4 (1.8)						4.4 (1.8)			4.6 (1.8)			4.2 (1.7)	0.008
Frequent	9,401			4.8 (1.7)						4.7 (1.7)			5.0 (1.8)			4.7 (1.8)	<0.001
*P* value		<0.001						<0.001			<0.001			<0.001			
Marital status, married																	
All	44,476	26,329	59.2		10,041	61.5		13,356	58.8		2,422	55.5		510	47.3		<0.001
Never/infrequent	18,743	12,091	64.5					10,882	64.3		1,070	67.0		139	64.4		0.094
Frequent	9,401	4,197	44.6					2,474	42.9		1,352	48.8		371	43.0		<0.001
*P* value		<0.001						<0.001			<0.001			<0.001			
Quantity of alcohol, g/day																	
All	44,476			5.4 (6.9)			0.7 (0.9)			5.2 (2.7)			16.8 (3.3)			34.6 (7.7)	<0.001
Never/infrequent	18,743			6.1 (5.2)						4.7 (2.6)			16.6 (3.2)			33.3 (6.8)	<0.001
Frequent	9,401			12.3 (9.3)						6.6 (2.6)			16.9 (3.3)			34.9 (7.92)	<0.001
*P* value		<0.001						<0.001			0.003			0.005			
Current smoker																	<0.001
All	44,476	13,465	30.3		4,438	27.2		6,967	30.7		1,551	35.5		509	47.2		
Never/infrequent	18,743	5,260	28.1					4,692	27.7		479	30.0		89	41.2		<0.001
Frequent	9,401	3,767	40.1					2,275	39.4		1,072	38.7		420	48.7		<0.001
*P* value		<0.001						<0.001			<0.001			0.057			
Physical activity, 1–4^e^																	
All	44,476			2.0 (0.8)			1.9 (0.7)			2.0 (0.8)			2.1 (0.8)			2.0 (0.8)	<0.001
Never/infrequent	18,743			2.0 (0.7)						2.0 (0.7)			2.0 (0.8)			1.9 (0.8)	0.122
Frequent	9,401			2.1 (0.8)						2.1 (0.8)			2.1 (0.8)			2.0 (0.8)	0.090
*P* value		<0.001						<0.001			0.014			0.062			
Body mass index^f^																	
All	44,476			25.9 (4.1)			26.1 (4.4)			25.7 (3.9)			25.7 (3.8)			25.9 (3.8)	<0.001
Never/infrequent	18,743			25.7 (3.9)						25.7 (3.9)			25.4 (3.6)			25.8 (3.7)	0.022
Frequent	9,401			25.8 (3.8)						25.8 (3.8)			25.8 (3.8)			25.9 (3.8)	0.442
*P* value		0.001						0.051			<0.001			0.634			
Diabetes																	
All	43,782	1,043	2.4		531	3.3		396	1.8		83	1.9		33	3.1		<0.001
Never/infrequent	18,445	364	2.0					319	1.9		38	2.4		7	3.3		0.153
Frequent	9,263	148	1.6					77	1.4		45	1.6		26	3.1		0.001
*P* value		0.032						0.007			0.097			1.000			
HDL-C, mmol/L																	
Women	18,933			1.58 (0.40)			1.52 (0.38)			1.62 (0.40)			1.76 (0.42)			1.80 (0.48)	<0.001
Never/infrequent	7,954			1.63 (0.41)						1.62 (0.40)			1.81 (0.44)			1.71 (0.43)	<0.001
Frequent	2,458			1.64 (0.39)						1.60 (0.38)			1.72 (0.40)			1.83 (0.49)	<0.00
*P* value		0.331						0.082			<0.001			0.152			
Men	21,122			1.33 (0.36)			1.26 (0.33)			1.33 (0.35)			1.42 (0.39)			1.48 (0.43)	<0.001
Never/infrequent	8,914			1.36 (0.37)						1.34 (0.36)			1.48 (0.40)			1.52 (0.48)	<0.001
Frequent	6,074			1.35 (0.36)						1.30 (0.32)			1.40 (0.38)			1.47 (0.41)	<0.001
*P* value		0.114						<0.001			<0.001			0.192			
Family history of CHD																	
All	44,476	17,566	39.5		6,675	40.9		8,846	39.0		1,643	37.6		402	37.3		<0.001
Never/infrequent	18,743	7,593	40.5					6,866	40.6		641	40.1		86	39.8		0.928
Frequent	9,401	3,298	35.1					1980	34.3		1,002	36.2		316	36.7		0.145
*P* value		<0.001						<0.001			0.010			0.436			

Abbreviations: CHD, coronary heart disease; HDL-C, high-density lipoprotein cholesterol; SD, standard deviation.

^a^ Frequent binge drinking (≥60 g of alcohol per occasion at least once per month) is also referred to as heavy episodic drinking. Never or infrequent binge drinking was defined as ≥60 g of alcohol per occasion less frequently than once per month.

^b^ Descriptive data are provided for participants with alcohol intakes < 2 g/day in the study population overall (“All”) but not in any stratum of binge-drinking frequency.

^c^ Analysis of variance and the χ^2^ test were used to assess for differences across categories.

^d^ Range of 1–8, in which 1 equals primary school and 8 a master’s or doctoral degree.

^e^ Range of 1–4, in which 1 is sedentary and 4 very active.

^f^ Weight (kg)/height (m)^2^).

### Incident IHD

The mean (standard deviation) follow-up time for IHD events in the study population was 9.0 (2.7) years and 1,535 events occurred (3.5%). Table 2 shows the number of events according to average drinking quantity and binge-drinking frequency. There was no clear and consistent difference in IHD risk between frequent binge drinkers and never or infrequent binge drinkers when the association was adjusted for or stratified by the average drinking quantity (Table 3). Stratified analyses by age (Web Table 2) showed a lower IHD risk among frequent binge drinkers younger than 50 years, but no variation was found according to sex (Web Table 3). Hazard ratios for IHD were consistently lower in groups reporting average intakes of 2.00–11.99 g/day and 12.00–23.99 g/day in comparison with the group drinking less than 2 g/day, regardless of whether they reported frequent or infrequent or never binge drinking (Table 4). Hazard ratios were also lower for those drinking 24.00–59.99 g/day in comparison with those drinking less than 2 g/day, but the confidence intervals were wide in the stratified analysis of infrequent or never binge drinkers. Including lifetime abstainers to the reference group did not materially change the results (Web Table 4).

**Table 2:** Number of Events for Incident Ischemic Heart Disease and Stroke According to Binge-Drinking Frequency and Average Drinking Quantity Among 44,476 Norwegian Adult Men and Women Who Participated in a Cardiovascular Health Examination Survey in Midlife and Reported to be Currently Drinking Alcohol, 1987–2003

	**Average Quantity of Alcohol Consumed, g/day**									
	**<2.00**		**2.00–11.99**		**12.00–23.99**		**24.00–59.99**		**2.00–59.99**	
**Binge-Drinking Frequency**	**No. of Events**	**Total No.**	**No. of Events**	**Total No.**	**No. of Events**	**Total No.**	**No. of Events**	**Total No.**	**No. of Events**	**Total No.**
*Incident Ischemic Heart Disease*										
All	706	15,626	649	22,050	144	4,223	36	1,042	829	27,315
Never/infrequent			544	16,386	75	1,522	14	202	633	18,110
Frequent			105	5,664	69	2,701	22	840	196	9,205
*Incident Stroke*										
All	547	15,785	598	22,101	132	4,235	57	1,021	787	27,357
Never/infrequent			507	16,423	80	1,517	25	191	612	18,131
Frequent			91	5,678	52	2,718	32	830	175	9,226

**Table 3:** Hazard Ratios With 95% Confidence Intervals for Incident Ischemic Heart Disease According to Binge-Drinking Frequency Among 44,476 Norwegian Adult Men and Women Who Participated in a Cardiovascular Health Examination Survey in Midlife and Reported to be Currently Drinking Alcohol, 1987–2003

	**Binge-Drinking Frequency** ^**a**^ ^**b**^							
	**Never/Infrequent**		**Frequent** ^**c**^		**Frequent** ^**d**^		**Frequent** ^**e**^	
**Average Quantity of Alcohol Consumed, g/day**	**HR**	**95% CI**	**HR**	**95% CI**	**HR**	**95% CI**	**HR**	**95% CI**
2.00–11.99	1.00		1.09	0.87, 1.36	0.98	0.78, 1.22		
12.00–23.99	1.00		0.82	0.58, 1.17	0.76	0.54, 1.08		
24.00–59.99	1.00		0.66	0.32, 1.33	0.61	0.29, 1.27		
2.00–59.99	1.00		0.97	0.82, 1.15	0.89	0.75, 1.06	0.91	0.76, 1.09

Abbreviations: CI, confidence interval; HR, hazard ratio.

^a^ Binge-drinking frequency was dichotomized into frequent (heavy episodic drinking; ≥60.00 g of alcohol per occasion at least once per month) and never or infrequent (≥60.00 g of alcohol per occasion less frequently than once per month).

^b^ Participants with average alcohol intake < 2.00 g/day were not included in the analyses.

^c^ HRs and 95% CIs were derived from Cox models that were adjusted for age and sex.

^d^ HRs and 95% CIs were derived from Cox models that were adjusted for age, sex, education, marital status, smoking, physical activity, body mass index, and familial history of coronary heart disease.

^e^ HRs and 95% CIs were derived from Cox models that were adjusted for age, sex, education, marital status, smoking, physical activity, body mass index, familial history of coronary heart disease, and the average quantity of alcohol consumed.

**Table 4:** Hazard Ratios With 95% Confidence Intervals for Incident Ischemic Heart Disease According to Average Quantity of Alcohol Consumed Among 44,476 Norwegian Adult Men and Women Who Participated in a Cardiovascular Health Examination Survey in Midlife and Reported to be Currently Drinking Alcohol, 1987–2003

	**All Participants**						**Binge-Drinking Frequency** ^**a**^ ^**b**^			
	**All** ^**c**^		**All** ^**d**^		**All** ^**e**^		**Never/Infrequent** ^**e**^		**Frequent** ^**e**^	
**Average Quantity of Alcohol Consumed, g/day**	**HR**	**95% CI**	**HR**	**95% CI**	**HR**	**95% CI**	**HR**	**95% CI**	**HR**	**95% CI**
2.00–11.99	1.00		1.00		1.00		1.00		1.00	
12.00–23.99	0.72	0.65, 0.80	0.75	0.67, 0.83	0.75	0.67, 0.84	0.75	0.67, 0.84	0.71	0.57, 0.88
24.00–59.99	0.70	0.59, 0.84	0.73	0.61, 0.88	0.76	0.62, 0.93	0.76	0.60, 0.97	0.68	0.52, 0.88
2.00–59.99	0.65	0.46, 0.91	0.61	0.43, 0.85	0.64	0.45, 0.91	0.79	0.46, 1.34	0.52	0.34, 0.80
2.00–11.99	0.71	0.64, 0.79	0.74	0.66, 0.82	0.75	0.67, 0.84	0.75	0.67, 0.84	0.67	0.56, 0.80

Abbreviations: CI, confidence interval; HR, hazard ratio.

^a^ Binge-drinking frequency was dichotomized into frequent (heavy episodic drinking; ≥60.00 g of alcohol per occasion at least once per month) and never or infrequent (≥60.00 g of alcohol per occasion less frequently than once per month).

^b^ Participants with alcohol intake < 2.00 g/day were used as the joint reference category in the stratified analyses according to binge-drinking frequency.

^c^ HRs and 95% CIs were derived from Cox models that were adjusted for age and sex.

^d^ HRs and 95% CIs were derived from Cox models that were adjusted for age, sex, education, marital status, smoking, physical activity, body mass index, and familial history of coronary heart disease.

^e^ HRs and 95% CIs were derived from Cox models that were adjusted for age, sex, education, marital status, smoking, physical activity, body mass index, and familial history of coronary heart disease, and/or stratified by binge-drinking frequency.

### Incident stroke

The mean follow-up time for stroke was 9.1 (2.7) years and 1,334 events occurred among the study population (3.0%). Table 2 shows the number of events according to average drinking quantity and binge-drinking frequency. There was not a clear and consistent difference in hazard ratios when comparing frequent binge drinkers and those who never or infrequently binge drink in analyses stratified or adjusted for the average drinking quantity (Table 5). Stratified analysis showed no clear variation by age (Web Table 2) or sex (Web Table 3). In analyses of average drinking quantity (Table 6), we used participants reporting low intakes (<2.00 g/day) as the reference group.

**Table 5:** Hazard Ratios With 95% Confidence Intervals for Incident Stroke According to Binge-Drinking Frequency Among 44,476 Norwegian Adult Men and Women Who Participated in a Cardiovascular Health Examination Survey in Midlife and Reported to be Currently Drinking Alcohol, 1987–2003

	**Binge-Drinking Frequency** ^**a**^ ^**b**^							
	**Never/Infrequent**		**Frequent** ^**c**^		**Frequent** ^**d**^		**Frequent** ^**e**^	
**Average Quantity of Alcohol Consumed, g/day**	**HR**	**95% CI**	**HR**	**95% CI**	**HR**	**95% CI**	**HR**	**95% CI**
2.00–11.99	1.00		1.29	1.02, 1.63	1.21	0.95, 1.53		
12.00–23.99	1.00		0.82	0.57, 1.19	0.81	0.56, 1.17		
24.00–59.99	1.00		0.54	0.31, 0.93	0.58	0.33, 1.02		
2.00–59.99	1.00		1.13	0.95, 1.35	1.07	0.90, 1.28	0.98	0.81, 1.19

Abbreviations: CI, confidence interval; HR, hazard ratio.

^a^ Binge-drinking frequency was dichotomized into frequent (heavy episodic drinking; ≥60.00 g of alcohol per occasion at least once per month) and never or infrequent (≥60.00 g of alcohol per occasion less frequently than once per month).

^b^ Participants with average alcohol intake < 2 g/day were not included in analyses.

^c^ HRs and 95% CIs were derived from Cox models that were adjusted for age and sex.

^d^ HRs and 95% CIs were derived from Cox models that were adjusted for age, sex, education, marital status, smoking, physical activity, body mass index, and familial history of coronary heart disease.

^e^ HRs and 95% CIs were derived from Cox models that were adjusted for age, sex, education, marital status, smoking, physical activity, body mass index, familial history of coronary heart disease, and the average quantity of alcohol consumed.

**Table 6:** Hazard Ratios With 95% Confidence Intervals for Incident Stroke According to Average Quantity of Alcohol Consumed Among 44,476 Norwegian Adult Men and Women Who Participated in a Cardiovascular Health Examination Survey in Midlife and Reported to be Currently Drinking Alcohol, 1987–2003

	**All Participants**						**Binge-Drinking Frequency** ^**a**^ ^**b**^			
	**All** ^**c**^		**All** ^**d**^		**All** ^**e**^		**Never/Infrequent** ^**e**^		**Frequent** ^**e**^	
**Average Quantity of Alcohol Consumed, g/Day**	**HR**	**95% CI**	**HR**	**95% CI**	**HR**	**95% CI**	**HR**	**95% CI**	**HR**	**95% CI**
2.00–11.99	1.00		1.00		1.00		1.00		1.00	
12.00–23.99	0.97	0.86, 1.09	0.99	0.88, 1.11	0.99	0.88, 1.12	0.97	0.85, 1.09	1.08	0.85, 1.37
24.00–59.99	1.00	0.82, 1.21	1.01	0.83, 1.23	1.04	0.84, 1.28	1.14	0.90, 1.44	0.84	0.62, 1.12
2.00–59.99	1.67	1.27, 2.20	1.58	1.20, 2.09	1.64	1.21, 2.22	2.17	1.45, 3.24	1.27	0.88, 1.84
2.00–11.99	1.00	0.90, 1.12	1.02	0.91, 1.14	1.01	0.90, 1.14	1.01	0.89, 1.13	1.02	0.84, 1.23

Abbreviations: CI, confidence interval; HR, hazard ratio.

^a^ Binge-drinking frequency was dichotomized into frequent (heavy episodic drinking; ≥60.00 g of alcohol per occasion at least once per month) and never or infrequent (≥60.00 g of alcohol per occasion less frequently than once per month).

^b^ Participants with intakes < 2.00 g/day were used as the joint reference category in the analyses stratified by binge-drinking frequency.

^c^ HRs and 95% CIs were derived from Cox models that were adjusted for age and sex.

^d^ HRs and 95% CIs were derived from Cox models that were adjusted for age, sex, education, marital status, smoking, physical activity, body mass index and familial history of coronary heart disease.

^e^ HRs and 95% CIs were derived from Cox models that were adjusted for age, sex, education, marital status, smoking, physical activity, body mass index, and familial history of coronary heart disease, and/or stratified by binge-drinking frequency.

In the overall analysis and in the stratified analysis of data from participants reporting never or infrequent binge drinking, hazard ratios for stroke were higher in the group reporting drinking 24.00–59.99 g/day in comparison with the reference group. Hazard ratios did not differ from the reference group among participants reporting drinking 2.00–11.99 g/day or 12.00–23.99 g/day. In the stratified analysis of data from participants reporting frequent binge drinking, there were no differences in the hazard ratio for stroke according to the average intake of alcohol. The inclusion of lifetime abstainers into a joint reference group did not materially alter the results (Web Table 4).

### Additional analyses

We repeated the analyses using drinking frequency instead of drinking quantity in a larger sample (*n* = 54,962). Hazard ratios for IHD events (Web Table 5) and stroke (Web Table 6) did not differ consistently or clearly between frequent and never or infrequent binge drinkers, but there were some single-group differences. Hazard ratios for IHD were lower in groups reporting a higher drinking frequency in comparison with infrequent drinking, regardless of binge-drinking status. Hazard ratios for stroke did not differ according to the drinking frequency. There was no clear difference in hazard ratios for IHD and stroke according to a more graded categorization of binge drinking when adjusted for average intakes of alcohol (Web Table 7).

## DISCUSSION

### Main findings

We hypothesized that the risk of major IHD and stroke events would be higher at a given alcohol intake level among frequent binge drinkers than among drinkers who never or infrequently binge drink; however, we found little support in the data. We also hypothesized that alcohol intake would not be associated with a lower risk of IHD among frequent binge drinkers. However, irrespective of binge-drinking frequency, the risk of IHD was lower among drinkers with an average intake of low to moderate quantities of alcohol (2.00–23.99 g/day) and, to a large extent, lower among drinkers with a higher intake (24.00–59.99 g/day), in comparison with drinkers with a low intake (<2.00 g/day). An increased risk of stroke at high alcohol intake levels was observed.

### Methodological considerations

Our aim for this study was to compare the risk of CVD between people who drink similar quantities of alcohol on average but differ in whether they tend to binge drink. To do this, we studied data from people who drink alcohol within a self-reported intake range that could be subjected to a fair comparison. Nondrinkers, therefore, were excluded—something that others have argued for when the aim was to compare different levels of drinking (3). People with an average drinking quantity of at least 60.00 g/day (i.e., heavy drinking) were also excluded, because, by definition, they binge drink every day, on average. People reporting low intakes (<2.00 g/day) also did not meet the study requirements, because one had to drink, on average, at least 2.00 g/day in order to binge drink once per month (≥60.00 g per occasion), but we included data from such people to act as a common reference group. It has been proposed that using low-intake drinkers together with lifetime nondrinkers is the least-biased reference group (21), but results did not change materially by including lifetime nondrinkers in the common reference group, or by including current nondrinkers and chronic heavy drinkers to the model.

The analyses of average drinking quantity in the study population overall showed a lower risk of IHD events at intakes of 2.00–59.99 g/day and higher risk of stroke at intakes between 24.00 and 59.99 g/day, in comparison with a low intake. This is in line with consortium individual-level data on current drinkers, reporting lower risk of MI in the intake range of 4.00 to 60.00 g/day, a slightly higher risk of stroke at intake up to approximately 20.00 g/day, and a markedly higher risk of stroke in the intake range of 20.00–60.00 g/day, in comparison with intakes less than approximately 3.50 g/day (3). The comparison with other data strengthens the internal and external validity of the findings.

Data on alcohol intake were based on a single measurement and the questionnaires did not inquire about changes in drinking habits prior to the survey. The inability to identify individuals who made drastic changes to their alcohol intake before or after the survey is a limitation. The hospital records used to identify events from IHD and stroke were available from 1994. This was 5 and 7 years prior to the examination date for 2 surveys, of which 1 was only part of the additional analyses involving drinking frequency. During this period, nonfatal events that occurred were unrecorded, which could lead to immortal time bias (i.e., time at risk with no possibility of an outcome). However, we checked that associations were in the same direction if the 2 surveys were excluded or analyzed separately.

Norway has a fairly low alcohol consumption per capita in comparison with many other countries, but at the same time, there is a high prevalence of binge drinking (16). This provides a good setting for a study designed to isolate the independent association of binge drinking with the risk of IHD and stroke. Less optimal settings include countries where binge drinking is rare and countries where frequent binge drinking often co-occur with chronic heavy drinking. We know from previous studies that the health survey participants tend to underestimate their alcohol intake, as judged by their HDL-C levels (22). This should be taken into account when considering the findings from this study.

### Interpretation

This study was performed using data from a population with no history of major IHD or stroke events. We identified 6 previous cohort studies of IHD (23–28) and a few previous studies (mix of methods) of stroke (29–33) in which the authors conducted relevant comparisons in populations defined as being free of CVD. Not all studies in a previous meta-analysis from 2008 were relevant (17): some included patients with CVD, some did not measure CVD status or did not clearly define the study population. The majority of the relevant studies reported risk estimates in direction of a greater risk of IHD and stroke among people who tend to binge drink. This includes an especially well-designed study of individuals in Northern Ireland and France who, on average, drank at least once per week, but less than 50 g/day (27). It also includes a US study (24) and a Danish study (25), for which risk estimates relevant to binge drinking were extracted and reported by the authors of the meta-analysis (17). Authors of a Finnish study found an increased risk of IHD and stroke associated with binge drinking among men without a previous MI and among men and women without a previous stroke, respectively (26, 31). Data in the direction of a higher risk of stroke associated with binge drinking were reported in 2 case-control studies (32, 33). Studies that did not find an increased risk associated with binge drinking include our study, a Danish study (28) of women and men adhering to sensible drinking guidelines (i.e., ≤14 and 21 drinks/week, respectively), and a UK study (23, 29) comparing weekend drinking pattern (≥6 units) with occasional drinking. An increased risk of stroke also was not found in the UK study.

The heterogeneity in the findings could be a product of differences in study population, operationalization of binge drinking, information about important confounders, and statistical analyses. We had information on many confounders frequently adjusted for in observational studies and we performed stratified analyses by sex and age (10). We did not have information about former drinking. This is a major limitation that, among other issues related to this particular study and the study design, could contribute to residual confounding and reverse causation. If we assume the study findings are generalizable, however, then a possible interpretation could be that binge drinking does not contribute negatively in early stages of atherosclerosis. In autopsy studies (summarized by Thomsen (34)), similar or lower levels of coronary atherosclerosis were reported among chronic heavy drinkers in comparison with control subjects, which, despite the limitations of this study and autopsy studies, is a tantalizing finding. It is important to emphasize that this interpretation does not rule out a role for binge drinking as a potential independent risk factor for IHD or stroke, which is supported by observational data from studies that have included people with a history of or established CVD (17, 22). It rather implies that such an effect could be mediated by mechanisms more potent in people with established disease. Nevertheless, the known short-term health effects of binge drinking provide ample reasons to encourage drinkers to avoid binge-drinking episodes. A reduction in binge drinking in the general population would help reduce the disease burden attributable to harmful alcohol use. Future studies may reveal binge drinking has long-term health effects on organs other than those included in the cardiovascular system.

## CONCLUSION

Among Norwegians with no history of major IHD and stroke events, we found comparable risks of IHD and stroke among people who drank the same quantity of alcohol over time but who differed in their tendency to binge drink. Intakes of low to moderate amounts of alcohol over time were associated with a lower risk of IHD in comparison with very little intake, even among those who reported frequent binge drinking. High intakes were overall associated with a higher risk of stroke.

## Supplementary Material

Web_Material_kwab063
