# Supplementary material for: Associations of Binge Drinking With the Risks of Ischemic Heart Disease and Stroke: A Study of Pooled Norwegian Health Surveys
Source: Am J Epidemiol. 2021 Mar 15;190(8):1592–603. doi: 10.1093/aje/kwab063 (PMC8489425; doi:10.1093/aje/kwab063)
Supplement: Web_Material_kwab063 [file Web_Material_kwab063.pdf]

Binge drinking and risk of ischemic heart disease and stroke: a study of pooled Norwegian health surveys

Eirik Degerud, Gudrun Høiseth, Jørg Mørland, Inger Ariansen, Sidsel Graff-Iversen, Eivind Ystrom, Luisa Zuccolo, Grethe S. Tell, and Øyvind Næss.

Table of contents:

- Web Figure 1: Flow-chart
- Web Tables 1-7:
  - Web Table 1: Characteristics of the surveys and alcohol questions
  - Web Table 2: HRs for IHD and stroke stratified by age
  - Web Table 3: HRs for IHD and stroke stratified by sex
  - Web Table 4: Sensitivity analysis: Lifetime non-drinkers in joint reference group with low-intake drinkers
  - Web Table 5: HRs for IHD using average drinking frequency instead of the average quantity of alcohol consumed
  - Web Table 6: HRs for stroke with average drinking frequency instead of the average quantity of alcohol consumed
  - Web Table 7: Sensitivity analysis: Binge drinking as categorical variable
- References

Web Figure 1.

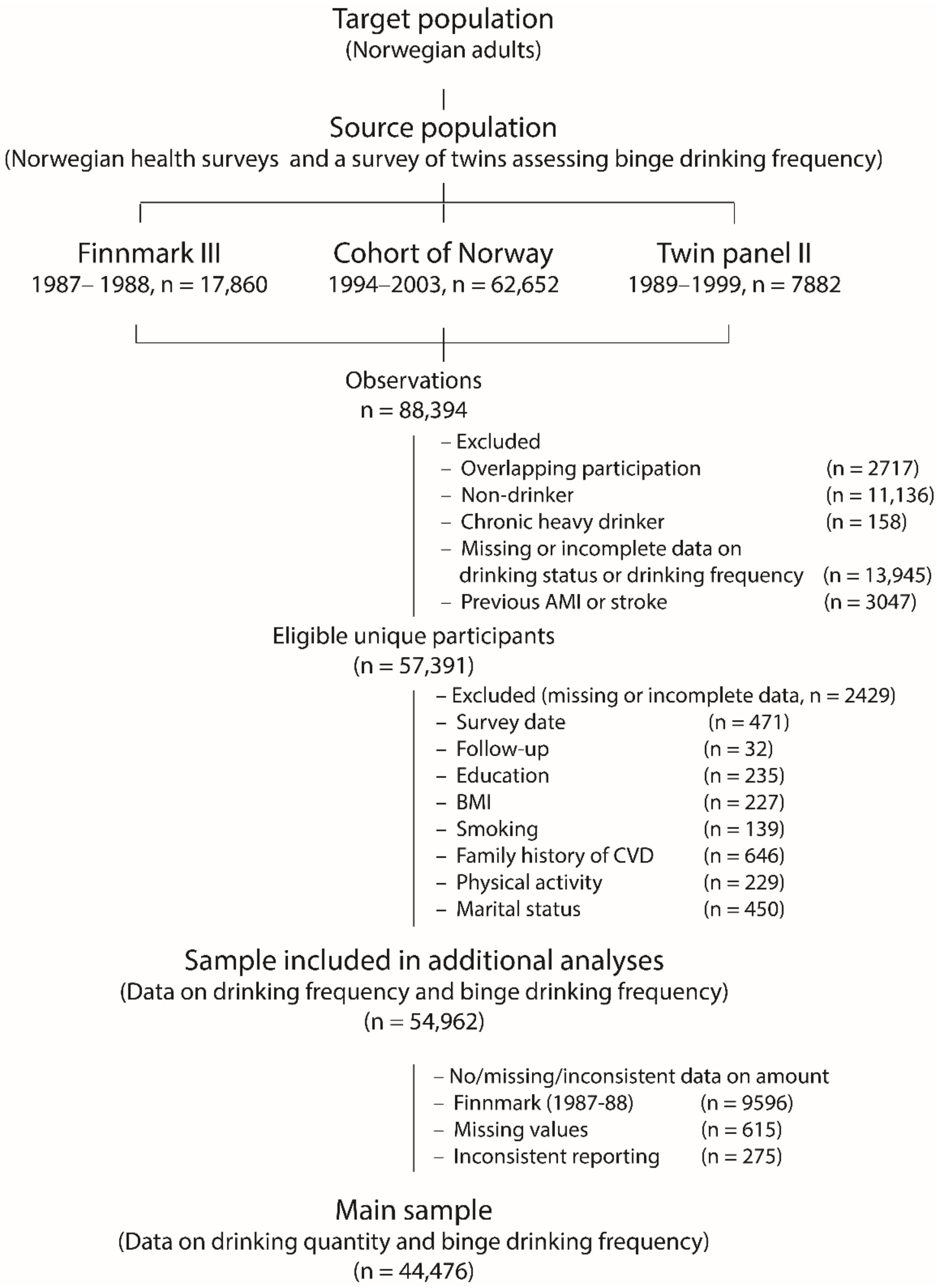

Web Figure 1. For the subgroup with data from more than one survey, we selected a single survey. Priority was a trade-off between the quality of alcohol data and the length of follow-up (Finnmark III > Cohort of Norway > Twin panel II). The excluded visits/observations are listed under “overlapping participation”.

Web Table 1. Characteristics and alcohol questions applied in the surveys that constituted the source population for this study.

	Survey year (range)	Participants (n)	Response rate (%)	Age (mean ± SD)	Age (range)	Sex (male	Average drinking frequency	Average drinking quantity	Binge drinking frequency
**The Counties Studies**									
Finnmark III	1987-1988	17,860	77.5	47.2 ± 9.3	(19 – 82)	50.6	A	NA	E
**Cohort of Norway (CONOR)**									
HUBRO	1996-2001	21,267	37.5	48.4 ± 15.5	(29.9 – 77.7)	44.5	B	C	F
I-HUBRO	2002	3526	29.9	38.8 ± 9.2	(19.2 – 60.9)	52.0	B	C	F
OSLO 2	1996-2001	6890	48.7	68.9 ± 6.2	(47.4 – 78.1)	100	B	C	F
OPPHED	2000-2001	12,210	55.5	50.1 ± 14.3	(29.9 – 76.8)	45.7	B	C	F
TROMSØ 5	2001	7833	78.0	59.8 ± 14.1	(29.5 – 88.9)	43.6	B	C	F
MoRo II	2003	1956	35.9	51.3 ± 9.7	(33.3 – 70.2)	44.4	B	C	F
REST-Troms	2002	2479	57.5	49.0 ± 13.8	(29.0 – 81.3)	43.6	B	C	F
REST-FINN	2002	6491	57.5	59.4 ± 11.0	(29.3 – 77.7)	47.6	B	C	F
**The Twin registry**									
Second panel	1989-1999	7882	83.7	49.4 ± 11.1	(30.0 – 79.0)	45.6	B	D	E*

Questions used to assess alcohol intake:

A) "How many glasses of beer, wine, or spirits do you usually drink during a two-week period?"
B) "How often during the past 12 months have you consumed alcohol?"
C) "How often during the past 12 months have you consumed alcohol" in combination with "When you drank alcohol, how many glasses did you usually drink?"
D) "How many glasses of beer, wine, or spirits do you usually drink during a week?"
E) "How often during the past year did you drink at least the equivalent of 5 small bottles of beer, a full bottle of wine (or ½ bottle of strong wine*) or a ¼ bottle of liquor?
F) Approximately how often during the past 12 months have you consumed alcohol corresponding to at least 5 glasses and/or spirits in 24 hours?
NA = not assessed

For more information about the surveys, please consult the following references for information: Cohort of Norway (1); Finnmark III (2); and the Twin registry second panel (3).

Web Table 2. The number of events and hazard ratios with 95% confidence intervals for incident ischemic heart disease and stroke according to binge drinking frequency in different age groups among 44,476 Norwegian adult men and women who participated in a cardiovascular health examination survey in midlife (1987 – 2003) and reported to be currently drinking alcohol.

Binge drinking frequency	Average drinking quantity 2 – <60 grams/day	
	Age <50 years (n = 17,118)	Age ≥50 years (n = 11,026)
**Ischemic heart disease**		
Event/no event (n)		
All	156/16,962	673/10,353
Never/infrequent	92/9921	541/8189
Frequent	64/7041	132/2164
HR (95% CI)		
Never/infrequent	1.00	
Frequent[1]	0.95 (0.69, 1.32)	0.97 (0.80, 1.18)
Frequent[2]	0.71 (0.51, 1.00)	0.94 (0.77, 1.14)
Frequent[3]	0.70 (0.49, 1.00)	0.97 (0.78, 1.20)
**Stroke**		
Event/no event (n)		
All	93/17,025	694/10,332
Never/infrequent	52/9961	560/8170
Frequent	41/7064	134/2162
HR (95% CI)		
Never/infrequent	1.00	
Frequent[1]	1.40 (0.91, 2.14)	1.09 (0.90, 1.32)
Frequent[2]	1.26 (0.81, 1.94)	1.04 (0.85, 1.26)
Frequent[3]	1.21 (0.76, 1.93)	0.94 (0.76, 1.17)

Binge drinking frequency was dichotomised into frequent (heavy episodic drinking; +60 grams of alcohol per occasion at least once per month) and never/infrequent (+60 grams of alcohol per occasion less frequent than once per month). Hazard ratios (HRs) and 95% confidence intervals (CIs) derived from Cox models adjusted for 1) age and sex; 2) plus education, marital status, smoking, physical activity, body mass index and familial history of coronary heart disease; 3) plus average drinking quantity.

Web Table 3. The number of events and hazard ratios with 95% confidence intervals for incident ischemic heart disease and stroke according to binge drinking frequency in strata of men and women who participated in a cardiovascular health examination survey in midlife (1987 – 2003) and reported drinking alcohol in the range of 2 – <60 g/day.

Binge drinking frequency	Average drinking quantity 2 – <60 grams/day	
	Women (n = 11.677)	Men (n = 16,467)
**Ischemic heart disease**		
Event/no event (n)		
All	128/11,549	701/15,766
Never/infrequent	111/8835	522/9275
Frequent	17/2714	179/6491
HR (95% CI)		
Never/infrequent	1.00	1.00
Frequent[1]	1.16 (0.68, 1.97)	0.94 (0.79, 1.12)
Frequent[2]	0.97 (0.56, 1.67)	0.88 (0.73, 1.05)
Frequent[3]	0.94 (0.53, 1.67)	0.90 (0.74, 1.09)
**Stroke**		
Event/no event (n)		
All	192/11,485	595/15,872
Never/infrequent	161/8785	451/9346
Frequent	31/2700	144/6526
HR (95% CI)		
Never/infrequent	1.00	1.00
Frequent[1]	1.56 (1.04, 2.33)	1.06 (0.87, 1.28)
Frequent[2]	1.41 (0.93, 2.12)	1.02 (0.83, 1.24)
Frequent[3]	1.27 (0.82, 1.96)	0.93 (0.75, 1.16)

Binge drinking frequency was dichotomised into frequent (heavy episodic drinking; +60 grams of alcohol per occasion at least once per month) and never/infrequent (+60 grams of alcohol per occasion less frequent than once per month). Hazard ratios (HRs) and 95% confidence intervals (CIs) derived from Cox models adjusted for [1)] age; [2)] plus education, marital status, smoking, physical activity, body mass index and familial history of coronary heart disease; [3)] plus average drinking quantity.

Web Table 4. The number of events and hazard ratios with 95% confidence intervals for incident ischemic heart disease and stroke according to average drinking quantity among 43,039 participants in the Cohort of Norway. Lifetime non-drinkers were included in the reference category together with low intake drinkers (<2 g/day). Current abstainers and chronic heavy drinkers were included in the models.

Binge drinking frequency	***Average drinking quantity (grams/day)***				
	<2 and lifetime non-drinkers	***2 – <12***	***12 – <24***	***24 – <60***	***2 – <60***
**Ischemic heart disease**					
Event/no event (n)					
All	620/13,996	550/20,032	117/3777	33/905	700/24,714
Never/infrequent	-	475/14,816	62/1344	13/165	550/16,325
Frequent	-	75/5216	55/2433	20/740	150/8389
Hazard ratio (95% CI) according to average drinking quantity					
All[1]	1.00	0.70 (0.63, 0.79)	0.66 (0.54, 0.81)	0.73 (0.51, 1.04)	0.70 (0.62, 0.78)
All[2]	1.00	0.73 (0.65, 0.82)	0.70 (0.57, 0.86)	0.68 (0.48, 0.97)	0.72 (0.64, 0.81)
Never/infrequent[2]	1.00	0.73 (0.65, 0.82)	0.71 (0.54, 0.92)	0.89 (0.51, 1.55)	0.73 (0.65, 0.82)
Frequent[2]	1.00	0.61 (0.47, 0.79)	0.62 (0.47, 0.83)	0.55 (0.35, 0.86)	0.61 (0.50, 0.74)
**Stroke**					
Event/no event (n)					
All	483/14,133	516/20,066	117/3777	49/889	682/24,732
Never/infrequent	-	442/14,849	73/1333	20/158	535/16,340
Frequent	-	74/5217	44/2444	29/731	147/8392
Hazard ratio (95% CI) according to average drinking quantity					
All[1]	1.00	0.96 (0.85, 1.09)	1.02 (0.83, 1.25)	1.72 (1.28, 2.32)	1.00 (0.89, 1.13)
All[2]	1.00	0.97 (0.86, 1.11)	1.03 (0.84, 1.27)	1.60 (1.19, 2.17)	1.01 (0.89, 1.14)
Never/infrequent[2]	1.00	0.95 (0.83, 1.08)	1.15 (0.90, 1.48)	2.12 (1.35, 3.32)	0.99 (0.87, 1.12)
Frequent[2]	1.00	1.11 (0.85, 1.44)	0.83 (0.60, 1.15)	1.34 (0.91, 1.98)	1.04 (0.85, 1.28)

Binge drinking frequency was dichotomised into frequent (heavy episodic drinking; +60 grams of alcohol per occasion at least once per month) and never/infrequent (+60 grams of alcohol per occasion less frequent than once per month). Hazard ratios (HRs) and 95% confidence intervals (CIs) derived from Cox models adjusted for [1)] age and sex; [2)] plus education, marital status, smoking, physical activity, body mass index and familial history of coronary heart disease. Participants with intakes <2 g/day and lifetime abstainers constituted the joint reference category. Current

abstainers (n = 2601) and chronic heavy drinkers (n = 104) were included in the overall models, but not in the models stratified by binge drinking frequency.

Web Table 5. The number of events and hazard ratios with 95% confidence intervals for incident ischemic heart disease according to binge drinking frequency and average drinking frequency among 54,962 participants in a cardiovascular health examination survey in midlife (1987 – 2003) who reported to be currently drinking alcohol.

Binge drinking frequency	***Average drinking frequency***				
	Infrequent	***1/month – 1/week***	***2 – 3/week***	***≥4 week***	***1/month – ≥4 week***
Event/no event (n)					
All	730/11,35[illegible]	1323/29,551	417/9051	128/2406	1868/41,008
Never/infrequent	–	1069/23,153	243/4981	78/1193	1390/29,327
Frequent	–	254/6398	174/4070	50/1213	478/11,681
HR (95% CI) according to binge drinking frequency[§]					
Never/infrequent	–	1.00	1.00	1.00	1.00
Frequent[1]	–	1.19 (1.03, 1.38)	1.10 (0.88, 1.36)	0.66 (0.45, 0.98)	1.02 (0.92, 1.14)
Frequent[2]	–	1.00 (0.86, 1.16)	0.95 (0.76, 1.18)	0.61 (0.41, 0.90)	0.90 (0.81, 1.01)
Frequent[3]	–	–	–	–	0.96 (0.85, 1.07)
HR (95% CI) according to average drinking frequency*					
All[1]	1.00	0.82 (0.75, 0.90)	0.68 (0.60, 0.77)	0.57 (0.47, 0.69)	0.77 (0.70, 0.84)
All[2]	1.00	0.86 (0.79, 0.95)	0.75 (0.66, 0.85)	0.65 (0.53, 0.78)	0.82 (0.75, 0.90)
All[3]	1.00	0.87 (0.79, 0.95)	0.76 (0.67, 0.87)	0.66 (0.54, 0.80)	0.84 (0.77, 0.92)
Never/infrequent[3]	1.00	0.86 (0.79, 0.95)	0.74 (0.64, 0.86)	0.74 (0.58, 0.94)	0.84 (0.76, 0.92)
Frequent[3]	1.00	0.83 (0.71, 0.97)	0.76 (0.64, 0.91)	0.54 (0.40, 0.73)	0.76 (0.67, 0.87)

Binge drinking frequency was dichotomised into frequent (heavy episodic drinking; +60 grams of alcohol per occasion at least once per month) and never/infrequent (+60 grams of alcohol per occasion less frequent than once per month). Hazard ratios (HRs) and 95% confidence intervals (CIs) derived from Cox models adjusted for [1)] age and sex; [2)] plus education, marital status, smoking, physical activity, body mass index and familial history of coronary heart disease; [3)] plus average drinking frequency or binge drinking frequency, as appropriate. [§]Infrequent drinkers (<once/month) were not included in analyses comparing binge drinking frequency directly, but used as the reference category in analyses of drinking frequency.

Web Table 6. The number of events and hazard ratios with 95% confidence intervals for incident stroke according to binge drinking frequency and average drinking frequency among 54,962 participants in a cardiovascular health examination survey in midlife (1987 – 2003) who reported to be currently drinking alcohol.

Binge drinking frequency	*Average drinking frequency*				
	Infrequent	*1/month – 1/week*	*2 – 3/week*	*≥4 week*	*1/month – ≥4 week*
**All events**					
Event/no event (n)					
All	510/11,576	988/29,886	369/9099	152/2382	1509/41,367
Never/infrequent	–	827/23,395	221/5003	88/1183	1136/29,581
Frequent	–	161/6491	148/4096	64/1199	373/11,786
HR (95% CI) according to binge drinking frequency §					
Never/infrequent	–	1.00	1.00	1.00	1.00
Frequent[1]	–	1.22 (1.02, 1.46)	1.42 (1.13, 1.79)	0.90 (0.63, 1.27)	1.24 (1.10, 1.41)
Frequent[2]	–	1.08 (0.90, 1.29)	1.29 (1.02, 1.63)	0.89 (0.62, 1.26)	1.13 (1.00, 1.28)
Frequent[3]	–	–	–	–	1.11 (0.97, 1.27)
HR (95% CI) according to average drinking frequency*					
All[1]	1.00	0.98 (0.88, 1.10)	1.00 (0.87, 1.15)	1.07 (0.89, 1.29)	1.00 (0.90, 1.10)
All[2]	1.00	1.02 (0.91, 1.14)	1.06 (0.92, 1.22)	1.15 (0.95, 1.39)	1.04 (0.93, 1.15)
All[3]	1.00	1.00 (0.90, 1.12)	1.02 (0.88, 1.19)	1.10 (0.90, 1.34)	1.01 (0.91, 1.13)
Never/infrequent[3]	1.00	1.01 (0.90, 1.13)	0.97 (0.82, 1.14)	1.11 (0.88, 1.40)	1.01 (0.90, 1.12)
Frequent[3]	1.00	1.02 (0.84, 1.24)	1.18 (0.97, 1.44)	1.15 (0.87, 1.51)	1.10 (0.95, 1.28)

Binge drinking frequency was dichotomised into frequent (heavy episodic drinking; +60 grams of alcohol per occasion at least once per month) and never/infrequent (+60 grams of alcohol per occasion less frequent than once per month). Hazard ratios (HRs) and 95% confidence intervals (CIs) derived from Cox models adjusted for 1) age and sex; 2) plus education, marital status, smoking, physical activity, body mass index and familial history of coronary heart disease; 3) plus average drinking frequency or binge drinking frequency, as appropriate. §Infrequent drinkers (<once/month) were not included in analyses comparing binge drinking frequency directly, but used as the reference category in analyses of drinking frequency.

Web Table 7. The number of events and hazard ratios with 95% confidence intervals for incident ischemic heart disease and stroke according to binge drinking frequency among 25,582 participants reporting binge drinking the last year in when attending a cardiovascular health examination survey in midlife (1987 – 2003).

		Binge drinking frequency		
	Not last year (not included)	A few times last year (n = 16,181)	1 – 3 times per month (n = 7420)	Once a week or more (n = 1981)
**Ischemic heart disease**				
Event/no event (n)		444/15,737	143/7277	53/1928
HR (95% CI)[1]	-	1.00	0.79 (0.65, 0.99)	1.04 (0.78, 1.38)
HR (95% CI)[2]	-	1.00	0.79 (0.65, 0.95)	0.97 (0.73, 1.30)
HR (95% CI)[3]	-	1.00	0.83 (0.68, 1.02)	1.03 (0.77, 1.39)
**Stroke**				
Event/no event (n)		332/15,849	138/7282	37/1944
HR (95% CI)[1]	-	1.00	1.12 (0.92, 1.37)	1.10 (0.78, 1.55)
HR (95% CI)[2]	-	1.00	1.11 (0.90, 1.35)	1.03 (0.73, 1.46)
HR (95% CI)[3]	-	1.00	1.13 (0.92, 1.40)	1.06 (0.75, 1.51)

Hazard ratios (HRs) and 95% confidence intervals (CIs) derived from Cox models adjusted for 1) age and sex; 2) plus education, marital status, smoking, physical activity, body mass index and familial history of coronary heart disease; 3) plus average drinking quantity.

References:

1. Naess O, Sogaard AJ, Arnesen E, Beckstrom AC, Bjertness E, Engeland A, et al. Cohort profile: cohort of Norway (CONOR). Int J Epidemiol. 2008;37(3):481-5.

2. Graff-Iversen S, Jansen MD, Hoff DA, Hoiseth G, Knudsen GP, Magnus P, et al. Divergent associations of drinking frequency and binge consumption of alcohol with mortality within the same cohort. J Epidemiol Community Health. 2013;67(4):350-7.

3. Magnus P, Berg K, Nance WE. Predicting zygosity in Norwegian twin pairs born 1915-1960. Clin Genet. 1983;24(2):103-12.
